# Multi-Omics Reveals the Effect of Crossbreeding on Some Precursors of Flavor and Nutritional Quality of Pork

**DOI:** 10.3390/foods12173237

**Published:** 2023-08-28

**Authors:** Qiangqiang Chen, Wei Zhang, Lixia Xiao, Qian Sun, Fen Wu, Guoliang Liu, Yuan Wang, Yuchun Pan, Qishan Wang, Jinzhi Zhang

**Affiliations:** 1 College of Animal Science, Zhejiang University, Hangzhou 310058, China; chenqiangqiang@zju.edu.cn (Q.C.); 21617003@zju.edu.cn (W.Z.); 22017065@zju.edu.cn (L.X.); 15340416220@163.com (Q.S.); 18805815950@163.com (F.W.); panyc@zju.edu.cn (Y.P.); wangqishan@zju.edu.cn (Q.W.); 2 Zhejiang Qinglian Food Co., Ltd., Jiaxing 314317, China; guoliangwuqi@163.com; 3 College of Animal Science and Technology, China Agricultural University, Beijing 100107, China; wangyuan@qau.edu.cn

**Keywords:** transcriptomics, metabolomics, pork, meat quality, longissimus dorsi muscle

## Abstract

Over the last several decades, China has continuously introduced Duroc boars and used them as breeding boars. Although this crossbreeding method has increased pork production, it has affected pork quality. Nowadays, one of the primary goals of industrial breeding and production systems is to enhance the quality of meat. This research analyzed the molecular mechanisms that control the quality of pork and may be used as a guide for future efforts to enhance meat quality. The genetic mechanisms of cross-breeding for meat quality improvement were investigated by combining transcriptome and metabolome analysis, using Chinese native Jiaxing black (JXB) pigs and crossbred Duroc × Duroc × Berkshire × JXB (DDBJ) pigs. In the longissimus Dorsi muscle, the content of inosine monophosphate, polyunsaturated fatty acid, and amino acids were considerably higher in JXB pigs in contrast with that of DDBJ pigs, whereas DDBJ pigs have remarkably greater levels of polyunsaturated fatty acids than JXB pigs. Differentially expressed genes (DEGs) and differential metabolites were identified using transcriptomic and metabolomic KEGG enrichment analyses. Differential metabolites mainly include amino acids, fatty acids, and phospholipids. In addition, several DEGs that may explain differences in meat quality between the two pig types were found, including genes associated with the metabolism of lipids (e.g., *DGKA*, *LIPG*, and *LPINI*), fatty acid (e.g., *ELOVL5*, *ELOVL4*, and *ACAT2*), and amino acid (e.g., *SLC7A2*, *SLC7A4*). Combined with the DEGS-enriched signaling pathways, the regulatory mechanisms related to amino acids, fatty acids, and phospholipids were mapped. The abundant metabolic pathways and DEGs may provide insight into the specific molecular mechanism that regulates meat quality. Optimizing the composition of fatty acids, phospholipids, amino acids, and other compounds in pork is conducive to improving meat quality. Overall, these findings will provide useful information and further groundwork for enhancing the meat quality that may be achieved via hybrid breeding.

## 1. Introduction

Over half of the world’s pork is produced in China, making it the country with the biggest pig industry [1]. The focus of pig breeding efforts over the last several decades has been to increase pig productivity in key areas such as feed conversion, growth rate, lean meat content, and lowered backfat thickness and amount of fat in the carcass. However, improvements in growth and carcass qualities have also influenced several characteristics of the meat’s quality, particularly intramuscular fat content, meat color, water-holding capacity, and tenderness [2]. The market share in China and worldwide is dominated by Duroc × Landrace × Yorkshire (DLY) pigs due to their superior feed conversion, rapid growth, and high leanness [2]. However, inferior DLY pork meat can elicit consumer dissatisfaction [3,4]. In contrast, Chinese local pork has a perfect color, aroma, and taste and is preferred by consumers, and these pigs are characterized by good reproductive performance and adaptability. However, native pig breeds are also associated with low leanness, low feed conversion rates, and high feeding costs; thus, pig farmers frequently prefer DLY pigs. In the past decades, mass production was pursued at the expense of quality. As the consumers’ quality demands have increased, the consumption of meat has increasingly evolved from “quantity” toward “quality”, and local pigs have gradually received more attention [4]. In recent years, crossbreeding of imported pig breeds as sires and native sows was found to produce progeny combining quality and quantity traits.

Notably, poor pork quality not only affects taste but also increases the risk of cardiovascular disease in humans [5]. Therefore, the molecular mechanisms involved in meat quality have attracted research attention. Currently, the quantitative trait loci (QTL) database of pigs (https://www.animalgenome.org/, accessed on 1 July 2023) comprises a large number of QTL that are related to the quality of the meat, e.g., 890 QTL associated with intramuscular fat content, 831 QTL associated with the saturated fatty acid content, and 750 QTL associated with monounsaturated fatty acid content. Most meat quality traits are complex quantitative traits, and many studies have focused on additive genetic effects, neglecting non-additive genetic effects. Non-additive effects will lead to nonlinear effects of phenotype. Understanding the mechanism of meat quality regulation will help researchers better understand the epistasis in non-additive effects. However, accurately determining the biomolecular mechanisms of meat quality is challenging, and new methods are warranted to assess meat quality traits in an accurate manner, thus improving productivity and meeting industry needs.

Multi-omics association analysis has been widely used in the fields of agricultural animal breeding [6,7,8]. The transcriptome can provide a comprehensive basis for elucidating processes of gene modulation transcriptionally; moreover, metabolomic analysis can inform on auxiliary metabolic changes caused by post-transcriptional modulation, which often represents a sequence of activities and alterations in the final stage of the gene transcription and translation [9]. In a previous study, transcriptome and targeted metabolome analyses were used to elucidate the effects of bile acids on fat deposition and meat quality of lambs [10]. In a different study, the transcriptome was used to mine key candidate genes regulating pork quality [11], and transcriptome analyses were applied to reveal the effects of regulatory mechanisms of myoglobin content on Iberian pork color [12]. Furthermore, transcriptomic data from porcine subcutaneous and intramuscular fat suggested new directions to study the regulatory mechanisms of two important adipose tissues in pigs [9]. These studies contribute to a better understanding of meat quality regulation mechanisms through multi-omics. Owing to its excellent and unique flavor, Jiaxing black (JXB) pork is very popular with consumers. Previous studies have shown that among the three breeds (JXB, Berkshire × JXB (BJ), and Duroc × Berkshire × JXB (DBJ)), DBJ pigs had the best carcass traits, while various aspects of JXB pigs’ meat quality were superior to those of DBJ and BJ pigs [13]. Therefore, from the perspective of economic efficiency and consumer demand, DBJ pigs can be used as a high-quality commercial breed, however, after several generations of breeding, DBJ pigs show segregation of hair color, which implies that the heritability of various traits of DBJ pigs is unstable. After we crossed Duroc sires with DBJ sows, trait heritability was stable in DDBJ pigs. Therefore, the meat quality of DDBJ and JXB pigs was analyzed comparatively, and comprehensive transcriptome and metabolome analyses were performed. The primary goal was to further optimize the meat quality of crossbred pigs with balanced genetic improvement and elucidate the potential genetic impacts of crossbreeding on improved pork quality.

## 2. Materials and Methods

### 2.1. Ethics Statement

Zhejiang University’s Animal Protection Institution and Use Committee (Zhejiang, Hangzhou, China) granted its approval to carry out experiments involving animals. The ethics committee number of this research institution is ZJU20170466.

### 2.2. Study Animals and Sample Collection

**Figure 1:**
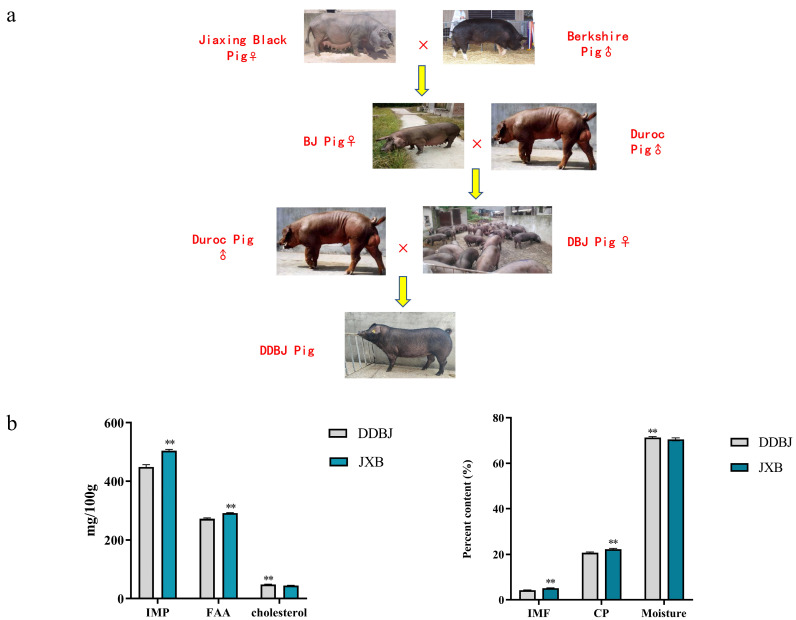
Crossbreeding methods and meat quality indexes. (**a**) Diagram of the hybridization mode of Jiaxing black, Berkshire, and Duroc pigs to produce DDBJ pigs. (**b**) The difference in inosinic monophosphate (IMP), free amino acid (FAA), cholesterol, intramuscular fat (IMF), crude protein (CP), and moisture in LD muscle between DDBJ pigs and JXB pigs; ** denotes significant difference at α = 0.01.

Pigs used in this study were provided by Zhejiang Qinglian Food Co. (Jiaxing, China), and were utilized in this investigation. ALL pigs were kept in a pig house under similar environmental conditions, were fed the same basal diet (see Supplementary Table S1), and had access to water ad libitum. ALL pigs were executed using 100 V, 12 S, and 70 HZ electrical stimulation. The farms are equipped with slaughterhouses and temperature control equipment to avoid transport stress and heat stress before slaughter. All pigs are slaughtered on the same day and in the same batch. A total of 20 JXB pigs and 23 DDBJ (Figure 1a) pigs of similar age (280 ± 5 days) were selected for slaughter. Longissimus dorsi (LD) muscle was harvested, immediately transferred into lyophilization tubes and preserved in liquid nitrogen.

### 2.3. Carcass Characteristics

The weight (kg) of each pig was recorded before slaughter. The left side of the carcass was weighed in kilograms (kg) after the viscera, hooves, head, and tail had been removed (kidneys and slab oil were kept). Carcass length, carcass slant length, and loin eye muscle area (length × width × 0.7) were measured, followed by the separation of bone, meat, skin, and fat from the left carcass by professional dissectors for weighing and calculating the respective percentage (%) of carcass weight.

### 2.4. Content of Inosine Monophosphate d, Intramuscular Fat, and Cholesterol

To determine inosine monophosphate (IMP), 0.4 g LD muscle was cut up and placed in liquid nitrogen for grinding. Subsequently, 20 mL of cold 5% perchloric acid was added, and the mixture solution was introduced into a volumetric flask with 5% perchloric acid to fix the volume. Following a 10 min incubation period, 20 mL of supernatant was removed, the pH value was set to 6.5 by adding 1.25 mol/L NaOH, and the mixture solution was then introduced into a volumetric flask and fixed with distilled water. Then, 1.5 mL of the mixture was placed in a centrifuge tube and centrifugated at 13,000× *g* rpm and 4 °C for half an hour. Subsequently, the HPLC (high-performance liquid chromatography) analysis was conducted by placing 1 mL of the supernatant in a chromatography bottle (Waters 515 HPLC, Waters, Milford, MA, USA). The following chromatographic conditions were applied: Waters AtlantisTM dC18 column (5 μm, Φ 4.6 × 250 mm); mobile phase: 50 mmol/L pH 6.5 ammonium formate buffer solution (containing 5% methanol); flow rate: 1 mL/min; column temperature: 25 °C; injection volume: 10 μL; UV detection wavelength: 254 nm; run time: 8 min for the standard working solution and 12 min for the sample extract. The IMP content of the sample (mg/g) was computed as per the corresponding values of peak areas of the two solutions using the equation Ci = Cs × Ai × V/As/m.

Where the IMP content within the sample is symbolized by Ci (mg/g), the IMP working solution concentration is denoted by “Cs” (mg/mL), the sample’s IMP peak area response is indicated by “Ai”, the IMP standard working solution peak area response value is denoted by the “As”, the total volume of the sample is denoted by V (mL), and the mass of the sample is indicated by m (g).

The content of intramuscular fat (IMF), crude protein (CP), water, and cholesterol was determined by the Zhejiang Feed and Animal Nutrition Laboratory, in compliance with the National Standard Technical Specification for Determination of Pig Muscle Quality (GB/T 9695.7-2008). Cholesterol content was determined using corresponding kits (Nanjing Jiancheng Institute of Biological Engineering, TG, A110-1-1; TC, A111-1-1).

### 2.5. Amino Acid and Fatty Acid Content

The LD muscle samples, weighing 100 mg, were digested in 5 mL of 6 mol/L HCl solution. Subsequently, the digested solution was then transferred to a volumetric flask and diluted to 50 mL. The filtered sample was passed through a 0.22 μm aqueous phase filter into a centrifuge tube. A 2 mL aliquot of the filtrate was placed in an evaporating dish and evaporated at 60 °C in a water bath. Then, 4 mL of 0.02 mol/L HCl solution was added, and the sample was dissolved. The samples were stored at 4 °C for analysis using an ion-exchange amino acid analyzer (L8900, Hitachi, Tokyo, Japan). 

For the fatty acid analysis, 150 mg of LD muscle samples were weighed and mixed with 4 mL of chloroacetic acid/anhydrous methanol (1:10, *v*/*v*), 1 mL of n-hexane, and 1 mL of internal standard solution. The mixture was heated in a water bath at 80 °C for 2.5 h. After cooling to room temperature, the solution was mixed with 5 mL of 7% K_2_CO_3_ solution and centrifuged at 1200× g for 5 min. The supernatant was transferred to vials for analysis using a gas chromatograph (Agilent 1200 Series, Agilent, Palo Alto, CA, USA). Prior to GC analysis, each sample was filtered through a 0.22 nm filter membrane (model 7890 A, Agilent Technologies, Palo Alto, CA, USA). Finally, the fatty acid concentrations were analyzed using GC ChemStation software; https://www.agilent.com/en/support/software-informatics/multiinstrumentsoftwarerev (accessed on 1 July 2023) (Agilent Technologies, Palo Alto, CA, USA).

### 2.6. RNA Sequencing

SAMN 19237330 is the accession number for our data uploaded to the NCBI. Accurate quantitative transcriptome sequencing analysis was performed by Beijing Allwegene Technology Co. (Beijing, China). The RNA sequencing data output included sample extraction, sample detection, library construction, library inspection, and up-sequencing. RNA purity was assessed using a Nanodrop device (Thermo Fisher Scientific Shanghai Co., LTD, Shanghai, China). Subsequently, an Agilent 2100 instrument (Agilent, Palo Alto, CA, USA) was used to measure the length of RNA fragments. Next, enrichment was performed with the aid of magnetic oligo (dT) beads, and mRNA was fragmented with the addition of a fragmentation buffer. Afterward, the first strand of complementary DNA (cDNA) was produced by the process of reverse transcription with the aid of base random primers (random hexamers), whereas the second strand of cDNA was produced with the addition of buffer, DNA polymerase I, and dNTPs. Subsequently, AMPure XP beads were used in the process of purifying the double-stranded cDNA. The double-stranded cDNA that had been purified was subjected to end repair, the addition of A, as well as the addition of splice. Thereafter, AMPure XP beads (Beckman Coulter, Shanghai Co., LTD, Shanghai, China) were employed to determine the fragment size of the double-stranded cDNA. The construction of cDNA libraries was accomplished by means of PCR amplification. The constructed libraries were quality screened before being 2 × 150-bp paired-end sequenced using an Illumina platform (Illumina, San Diego, CA, USA). Comparison and transcript splicing analyses were completed using Star (http://star.mit.edu/ accessed on 1 July 2023) and Cufflinks version 2.2.0 software programs, respectively, followed by quantitative analysis of all genes.

### 2.7. Metabolome Analysis

In order to avoid the interference of the weight of the pigs on the experimental results, we selected 10 pigs (5 JXB pigs and 5 DDBJ pigs, as they have similar weights). Our study features a selection of five DDBJ and five JXB pigs, chosen as representative exemplars of their respective breeds’ mean values for carcass traits and meat quality attributes. The LD muscle samples were extracted from the −80 °C freezer and slowly thawed at 4 °C. An appropriate amount of sample was taken and added to a pre-cooled MeOH: ACN: H_2_O solution (v:v:v = 2:2:1) containing internal standards and two steel beads. The tissue homogenization was carried out using a tissue grinder at 60 Hz for 120 s, followed by sonication for 10 min. The mixture was allowed to stand at 20 °C for 1 h and then centrifuged at 13,000× g rpm for 15 min at 4 °C. The upper clear liquid was collected and freeze-dried. For mass spectrometry analysis, an appropriate amount of ACN: H_2_O solution (v:v = 1:1) was added to the dried samples, and the mixture was vortexed for 30 s and sonicated for 10 min. The solution was then centrifuged at 13,000× g rpm for 15 min at 4 °C. The upper clear liquid was transferred to an injection vial for subsequent LC-MS/MS analysis.

Extensive untargeted metabolome sequencing analysis was performed by Beijing Allwegene Technology Co. Ten samples were selected and were assigned to two groups for metabolic analyses based on extensively untargeted metabolomics performed using ultra-performance liquid chromatography (UPLC)–tandem mass spectrometry (MS/MS)-based detection platform, a self-built database, and multivariate statistics. The mobile phase for LC-MS/MS analysis consisted of eluent A (0.1% formic acid in water) and eluent B (0.1% formic acid in methanol). A Waters ACQUITY UPLC HSS T3 C18 column (1.8 μm, 2.1 mm × 100 mm) was used for reversed-phase separation. The injection volume was 2 μL, the flow rate was set at 0.3 mL/min, and the column temperature was maintained at 50 °C. The metabolites from the column were subjected to high-resolution mass spectrometry using Triple TOF 5600+ in both positive and negative ion modes. The detailed parameters were as follows: Ion Source Gas1: 50 psi, Ion Source Gas2: 50 psi, curtain gas: 35 psi, source temperature: 500 °C, IonSapary voltage floating: 5500 V and - 4500 V (positive and negative); declustering potential (DP): ±80 V (positive and negative); TOF MS scan *m*/*z* range: 60–1200 Da, product ion scan *m*/*z* range: 25–1200 Da, TOF MS scan accumulation time 0.25 s/spectra, product ion scan accumulation time 0.03 s/spectra. Two-stage mass spectrometry was acquired using information-dependent acquisition (IDA) in high sensitivity mode, with CE: 30 V ± 15. Data acquisition was performed using UPLC (ExionLC AD, https://sciex.com.cn/, accessed on 6 November 2022) and MS/MS (QTRAP^®^, https://sciex.com.cn/, accessed on 6 November 2022). Using the in-house created database of targeted standards, qualitative analysis was carried out on the substances that were discovered by using retention time (RT), daughter ion pair data, and secondary spectral data. The program known as Analyst 1.6.3 was used in the processing of the mass spectrometry data.

### 2.8. Evaluation of Variation in Gene Expression and Metabolic Profiles

The differential gene criterion was a q-value ≤ 0.05. The criteria for differential metabolites were VIP ≥ 1 and fold change ≤0.5 and ≥2. Differential gene ontology (GO) enrichment analysis was conducted using GO seq, which uses the Wallenius non-central hypergeometric distribution. The KEGG database’s pathways were used in a hypergeometric test-based differential gene KEGG enrichment study.

The KEGG Compound database (http://www.kegg.jp/kegg/compound/, accessed on 23 November 2022) was used for the annotation of the metabolites that were found before mapping them to the KEGG Pathway database (http://www.kegg.jp/kegg/pathway.html, accessed on 17 December 2022). Hypergeometric tests were conducted to examine the significance of the pathways to which the substantially regulated metabolites were mapped before processing them via a metabolite set enrichment analysis.

### 2.9. Data Analyses

We used GraphPad Prism 9.0 (GraphPad Software, San Diego, CA, USA) to analyze the variations in the contents of amino acids and fatty acids in the two pig breeds LD muscle tissue. Student’s *t*-test was applied for variance analysis when the variances were equal, and otherwise, Welch’s test was used. All data from the study are shown as means ± SEM (23 DDBJ pigs, 20 JXB pigs).

Using multiple hypothesis tests to correct *p*-value (FDR correction with Benjamin/Hochberg), the FDR values were obtained for screening DEGs. Based on the OPLS-DA results, the obtained multivariate analysis of the variable importance in projection (VIP) of the OPLS-DA model can preliminarily screen metabolites with differences between different varieties. *p*-value can be combined with univariate analysis (comparison between two groups: Student’s *t* test) (n = 5). We performed Pearson’s and Spearman correlation analyses of variance for differential genes enriched in the relevant pathways with the corresponding differential metabolites. Correlations were determined with the cor function in R, whilst correlation heat maps were produced using the Hmisc package in R (https://cran.r-project.org/web/packages/Hmisc/index.html, accessed on 6 January 2023). Subsequently, network plots were produced using Cytoscape software https://cytoscape.org/ (accessed on 1 July 2023) [14].

## 3. Results

### 3.1. Quality and Carcass Characteristics of JXB and DDBJ Pigs

**Figure 2:**
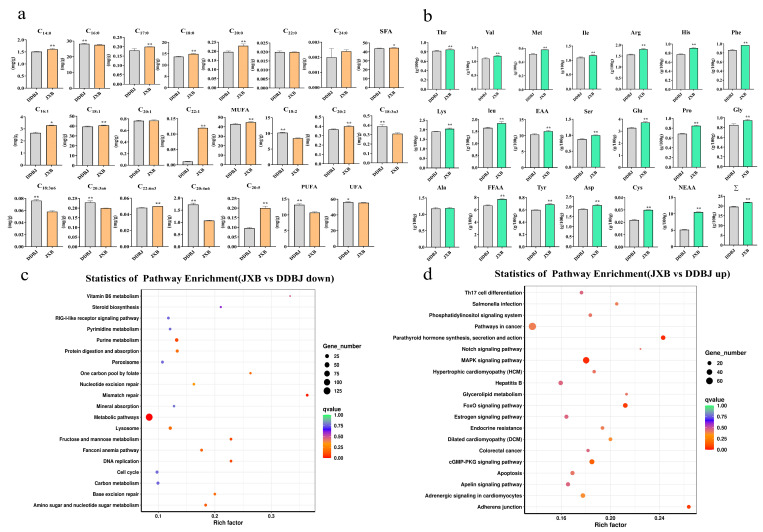
Comparison of fatty acid and amino acid levels in LD muscle of DDBJ and JXB pigs, and KEGG enrichment analysis. (**a**) Comparison of 19 fatty acids in LD muscle of DDBJ and JXB pigs. SFA = saturated fatty acid; UFA = unsaturated fatty acid; MUFA = monounsaturated fatty acid; PUFA = polyunsaturated fatty acid; SFA = C14:0 + C16:0 + C17:0 + C18:0 + C20:0 + C22:0 + C24:0, UFA = C16:1 + C18:1 + C18:2 + C18:3n6 + C18:3n3 + C20:1 + C20:2 + C20:3n6 + C20:4n6 + C20:5 + C22:1 + C22:6 n3; MUFA = C16:1 + C18:1 + C20:1 + C22:1; PUFA = C18:2 + C18:3n6 + C18:3n3 + C20:3n6 + C20:4n6 + C20:5. (**b**) Comparison of 17 amino acid levels in LD muscle of DDBJ and JXB pigs. EAA = essential amino acids, EAAΣ = Thr + Val + Met + Ile + Leu; FFAA = fresh flavor amino acid, FFAAΣ = Ser + Glu + Pro + Gly + Ala, NEAA = non-essential amino acid, NEAA = Asp + Cys + Tyr, Σ = total amino acid. Asterisk denotes significant difference; * α = 0.05, ** α = 0.01. (**c**,**d**) KEGG pathway enrichment, up- and downregulated differentially expressed genes (DEGs); shown are the first 20 pathways.

Pigs of the JXB breed had LD muscles with elevated levels of inosine monophosphate, free amino acids, intramuscular fat, and crude protein in contrast with those of the DDBJ breed (*p* < 0.01, Figure 1b). In addition, when comparing DDBJ with JXB pigs, it was found that the former had remarkably greater cholesterol and water levels (*p* < 0.01, Figure 1b). Additionally, JXB pigs exhibited considerably greater levels of monounsaturated fatty acids compared to DDBJ pigs, particularly in terms of C16:1 (*p* < 0.05), C18:1 (*p* < 0.01), and C22:1 (*p* < 0.01) (Figure 2a). By contrast, polyunsaturated fatty acid levels were substantially reduced in JXB relative to DDBJ pigs (*p* < 0.01), where C18:2, C18:3n3, C18:3n6, and C20:3n6 levels were remarkably elevated in DDBJ in contrast with JXB pigs (*p* < 0.01) and C20:2, C22:6n3, and C20:5 levels were remarkably elevated in JXB in contrast with DDBJ pigs (*p* < 0.01). Notably, unsaturated fatty acid levels were elevated in DDBJ as opposed to JXB pigs (Figure 2a). The carcass traits of DDBJ pigs were better than those of JXB pigs; specific carcass traits were compared in the Supplementary Materials.

The levels of essential amino acids (EAA) and non-EAA (NEAA) were considerably elevated in JXB in contrast with those of DDBJ pigs (*p* < 0.01). JXB pigs also had a remarkably higher level of fresh flavor amino acids compared to DDBJ pigs (*p* < 0.01). Figure 2b depicts the variations in the contents of 17 specific amino acids. We also compared several carcass traits of the two pig lines (Supplementary Figure S1).

### 3.2. Differentially Expressed Genes (DEGs) in LD Muscle Tissue

Our analysis revealed 2959 DEGs, including 1494 and 1465 up- and downregulated genes, respectively (Supplementary Figure S2a). Furthermore, KEGG enrichment classification showed predominant enrichment of DEGs in metabolism, genetic information processing, organic systems, environmental information processing, and cellular processes, among which KEGG pathways of the metabolism branch were the most enriched. The differential genes were enriched as follows: 33 in the nucleotide metabolism-associated pathways, 68 in the metabolism of cofactors and vitamin-related pathways, 75 in lipid metabolism-associated pathways, 112 in glycan biosynthesis and metabolism-associated pathways, 10 in energy metabolism-associated pathways, 53 in carbohydrate metabolism-associated pathways, and 67 in amino acid metabolism-associated pathways (Supplementary Figure S2b). The functional entries annotated by GO enrichment were divided into biological processes, cellular components, and molecular functions, and the GO functional terms under these three categories are displayed in Supplementary Figure S2c,d. In addition, we used the KEGG database to determine which pathways were enriched for the up and downregulated DEGs in the JXB and DDBJ pigs (Figure 2c,d). The downregulated genes showed primary enrichment in metabolic pathways, vitamin B6 metabolism, purine metabolism, protein digestion and absorption, mannose and fructose metabolism, nucleotide sugar and amino sugar metabolism, and other pathways. The upregulated genes were primarily enriched in the parathyroid hormone synthesis, secretion, and action, FoxO signaling pathway, MAPK signaling pathway, and the adherens junction pathway.

### 3.3. LD Muscle Metabolome Analysis

**Figure 3:**
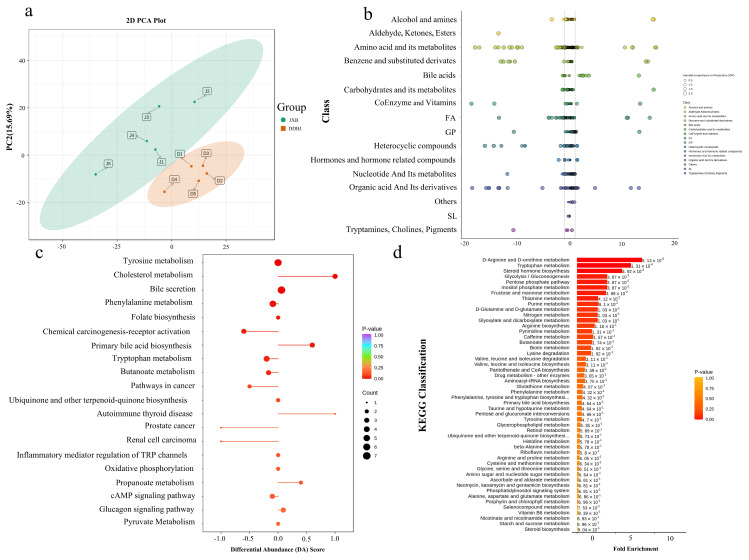
Metabolic analysis results of LD muscle in DDBJ and JXB pigs (**a**) Grouping principal component analysis; PC1 represents the first principal component, PC2 represents the second principal component, and the percentage represents the interpretation rate of the principal component to the dataset; each point in the figure represents a sample, samples of the same group are indicated by the same color, and “Group” is a group. (**b**) Differential metabolite scatter diagram, where each point represents a metabolite, and different colors represent distinct classifications. The abscissa denotes the logarithm (log_2_FC) of the relative content difference multiple of a substance in two sample groups. The size of the dot signifies the VIP value. (**c**) For the overall change analysis of the KEGG metabolic pathway, the ordinate represents the name of different pathways (sorted by *p*-values), and the abscissa indicates the DA score. The DA Score is the sum of all metabolite alterations throughout the metabolic pathway. An expression score of 1 shows an upregulation trend in the expression of all metabolites in this pathway, and a score of −1 signifies a downregulation trend in the expression of all of the detected metabolites within this pathway (**d**) Metabolite sets enrichment analysis; the ordinate highlights the name of the metabolic set (sorted by *p*-values), corresponding to the *p*-value of the metabolic set; the abscissa indicates fold enrichment.

The grouped samples were subjected to principal component analysis (PCA) to test variability between groups and among samples within each group (Figure 3a). We classified differential metabolites (Figure 3b, Supplementary Figure S3b–d). The top twenty pathways ranked by *p*-value from our KEGG pathway enrichment study of the differential metabolites were chosen (Supplementary Figure S4a). Similarly, the top 20 metabolic pathways according to *p*-value were chosen for examination of total KEGG metabolic pathway alterations, and the differential enrichment score was a reflection of the total alterations that occurred in all of a pathway’s metabolites (Figure 3c). Metabolic enrichment analysis indicated significant enrichment of the top 50 metabolic sets ranked by *p*-value, including D-arginine and D-ornithine metabolism, tryptophan metabolism, steroid biosynthesis, glycolysis/gluconeogenesis, inositol phosphate metabolism, mannose and fructose metabolism, thiamine metabolism, and pentose phosphate pathways (*p* < 0.05; Figure 3d). In addition, we performed HMDB functional annotation and enrichment of differential metabolites and found that they were significantly enriched in pathways such as thiamine metabolism, bile acid biosynthesis, and oxidation of branched-chain fatty acids (Supplementary Figure S4c).

### 3.4. Combined Transcriptomic and Metabolomic Analysis

**Figure 4:**
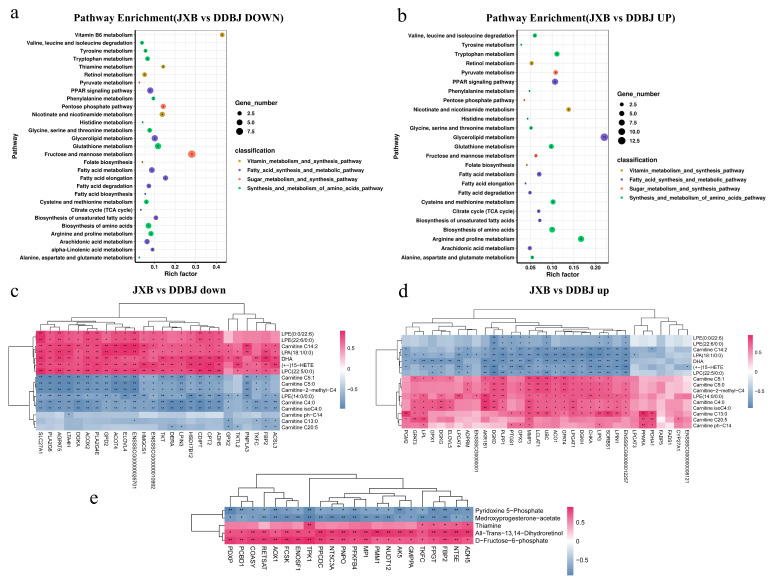
The enrichment analysis of DEGs in meat quality regulation-related pathways and correlation analysis between DEGs and differential metabolites (Spearman correlation). (**a**,**b**) Downregulation and upregulation of DEGs in KEGG enrichment pathway map; different colors represent different classified pathway sets, and bubble size indicates the number of DEGs. (**c**–**e**) Correlation analysis of DEGs and differential metabolites. (**c**,**d**) Spearman correlation analysis of DEGs with phospholipid metabolite and fatty acyl differential metabolite. (**e**) Spearman correlation analysis of DEGs and differential metabolite such as vitamin coenzyme and carbohydrate related to sugar metabolism and vitamin metabolism, color depth represents correlation strength, positive and negative correlation are denoted by red and blue colors, correspondingly; asterisks denote the significant variation * α = 0.05, ** α = 0.01.

We divided the list of meat quality-related KEGG pathways enriched through downregulated genes into three categories: synthesis and metabolism of amino acids pathways, fatty acid synthesis, and metabolic pathways, and sugar metabolism and synthesis pathways (Figure 4a). Among the meat quality-related KEGG pathways enriched through upregulated genes, four categories were identified: vitamin metabolism and synthesis pathways, fatty acid synthesis and metabolic pathways, synthesis and metabolism of amino acids pathways, and sugar metabolism and synthesis pathways (Figure 4b). The up- and downregulated differential genes in KEGG pathways related to fat metabolism and synthesis were subjected to Spearman correlation analysis with fatty acyl and glycerophospholipid metabolites, respectively (Figure 4c,d). The results showed that the downregulated genes *SLC27A1*, *PLA2G6*, *AGPAT5*, *LTA4H*, *DGKA*, *ACOT4*, *ACOX2*, *PLA2G4E*, *LPIN3*, *ELOVL4*, *ENSSSCG00000026701* and others were significantly and positively correlated with unsaturated glycerophospholipid metabolites such as: lysophosphatidylcholine (LPC (22:5/0:0)), lysophosphatidylethanolamine (LPE (0:0/22:6)), LPE (22:6/0:0)), and lysophosphatidic acid (LPA (18:1/0:0)). These were significantly negatively correlated with saturated glycerophospholipid metabolites (LPE (14:0/0:0)). The upregulated genes *AKR1B1*, *DGKD*, *PLPP1*, *LIPG*, *SORS1*, *CHKA*, *GPAT4*, *LCLAT1*, *LPGAT1*, *DGKH*, *ACO1*, *MMP3*, and *UBC* were significantly and positively correlated with carnitine C5:1, carnitine C5:0, carnitine-2-methyl-C, LPE (14:0/0:00), carnitine C4:0, and carnitine isoC4:0; the *DGKD*, *PLPP1*, *PTGS1*, *LIPG*, *SORBS1*, *ENSSSCG00000012257*, *MMP3*, *UBC*, and *GPX3* genes were significantly negatively correlated with carnitine C14:2, LPA(18:1/0:0), (+-)15-HETE, LPC(22:5/0:0), LPE(0:0/22:6), and LPE(22:6/0:0).

**Figure 5:**
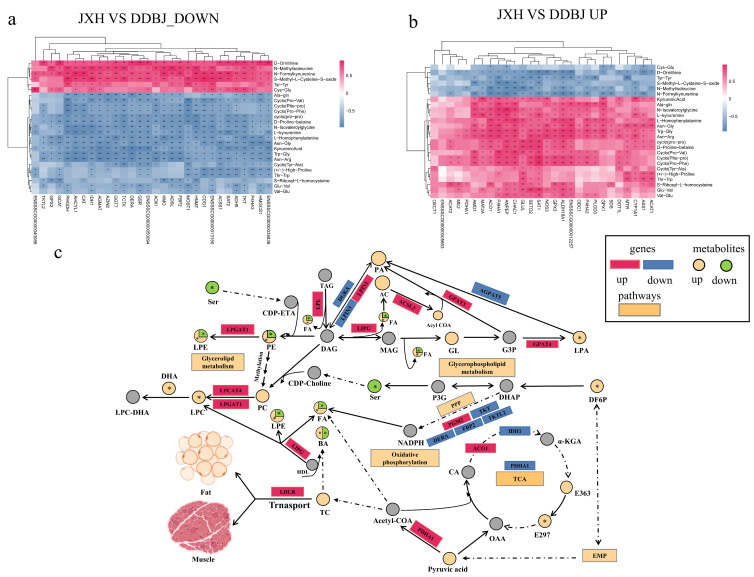
Spearman correlation analysis of DEGs and small peptides in the pathway linked to amino acid synthesis and metabolism and the regulatory mechanism of compound metabolism. (**a**,**b**) Spearman correlation analysis of up- and downregulated DEGs and small peptides. (**c**) The regulation mechanism diagram of KEGG pathway enriched DEGs combined with phenotypic data and differential metabolites. Genes are indicated by rectangles (|log_2_ (fold change)| > log_2_1, *p*_adj_ < 0.05), red and blue colors denote upregulated and downregulated genes, correspondingly. The yellow rectangle denotes the pathway, and the circle with * represents the differential metabolite (VIP > 1, |log2 (fold change)| > 2, *p*_adj_ < 0.05). The yellow circle represents upregulation, green represents downregulation, the alternating yellow and green represents both up- and downregulation of such substances, the gray represents no detection, and the asterisk indicates significant differences; the dotted line represents an indirect reaction, while the solid line does not necessarily represent a direct reaction, but may also represent reaction conditions and directions. Abbreviations: SAM-e: s adenosylmethionine, BA: bile acid, TC: cholesterol, HDL: high-density lipoprotein, EMP: glycolysis, PA: phosphatic acid, FA: fatty acid, TAG: triglyceride, LPA: lysophosphatidic acid, LPG: lysophosphatidylcholine, LPE: lysophosphatidylethanolamine, TAG: triglyceride, DAGS: diacylglycerol, MAG: monoglyceride, GL: glycerol, G3P: 3, glycerol phosphate, P3G: triglyceride triphosphate, DHA: dihydroxyacetone phosphate, DF6P: 6, fructose phosphate, α-KGA: α-Ketoglutarate, E363: succinic acid, E297: fumaric acid, OAA: oxaloacetic acid, CA: citric acid EMP: glycolysis, PPP: pentose phosphate pathway, PC: phosphatidylcholine, PE: phosphatidylethanolamine.

We compared the up- and downregulated differential genes in KEGG pathways associated with amino acid metabolism and synthesis with those of amino acids, amino acid derivatives, and small peptide metabolites by Spearman correlation analysis. Most downregulated genes had a substantial negative correlation with amino acids and small peptide metabolites, and most upregulated genes were shown to have a substantial positive association with amino acids and small peptide metabolites (Figure 5a,b). We also tested correlations of differential genes in pathways related to glucose metabolism and vitamin coenzyme metabolism with metabolites (Figure 4e). Genes including *TPK1*, *PDXP*, *COASY*, *AOX1*, *FCSK*, *ENOSF1*, *AK5*, *ADH5*, *TKFC*, and *PMM1* were significantly correlated with d-fructose-6-phosphate and all-trans-13,14-dihydroretinol, among which *TKFC*, *FPGT*, *FBP2*, *NT5E*, and *ADH5* were also significantly correlated with the thiamine content. The *AOX1* gene in the retinol metabolism pathway was positively correlated with the metabolite all-trans-13,14-dihydroretinol. The *TPK1* gene in the thiamine metabolism pathway was significantly positively correlated with the thiamine content.

### 3.5. Potential Regulatory Mechanisms and Functions Related to Meat Quality Hub Genes

**Figure 6:**
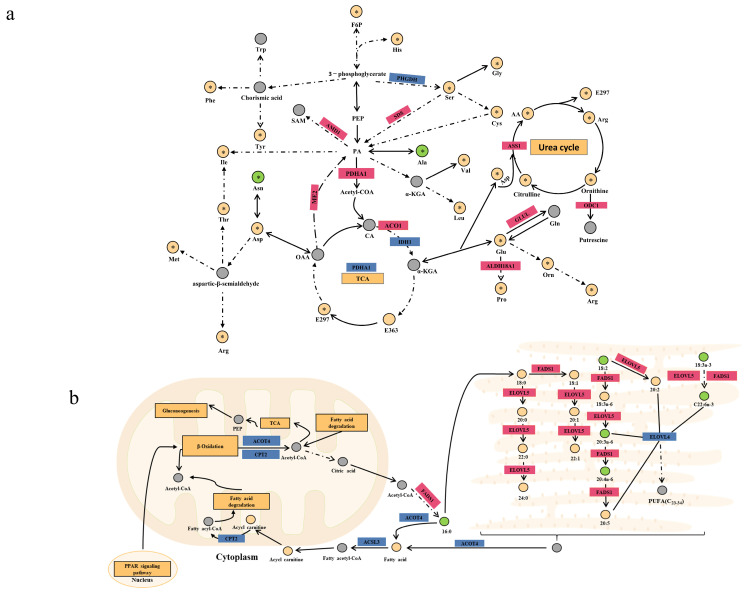
Regulation mechanism diagram of DEGs and differential metabolites (fats and amino acids). (**a**) Amino acid anabolism regulation network diagram. (**b**) Fatty acid anabolism regulation diagram. AA: argininosuccinic acid. The yellow rectangle represents the regulatory pathway, the red and blue rectangles denote the upregulated and downregulated genes, correspondingly; the yellow and green circles denote the upregulated and downregulated metabolites correspondingly, the circle with * represents the differential metabolite, and the gray signifies the undetected metabolite.

**Figure 7:**
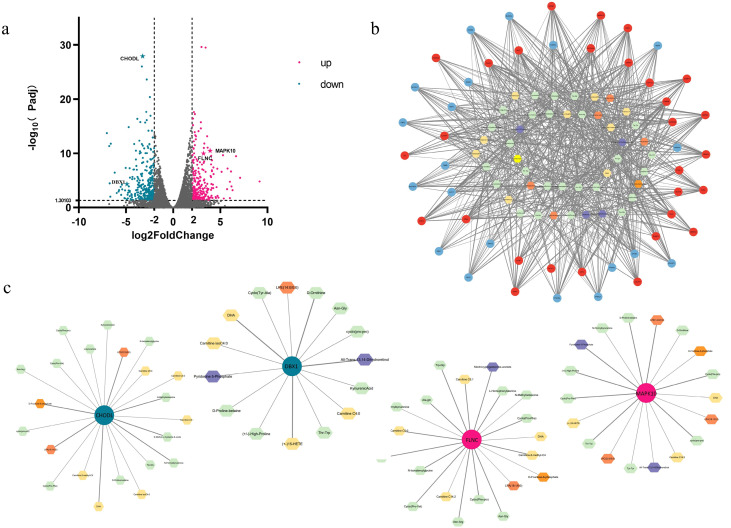
Network diagram of DEGs and differential metabolites. (**a**) With *p* < 0.05, |log2 (fold change)| > 2 as the screening condition, the discovered differential genes included 217 upregulated and 227 downregulated genes. (**b**) The specific correlation coefficient and *p*-value of the differential gene metabolite correlation network are shown in the supply tables. (**c**) According to correlation analysis, four target genes and their related networks with metabolites were selected. Small circles represent differential genes, blue and red denote downregulated and upregulated DEGs, correspondingly, the hexagon represents differential metabolites, and different colors represent different types of differentials.

After reviewing the literature and identifying relevant signaling pathways associated with the differential genes, we produced a differential gene metabolite regulation map based on phenotypic data and metabolome data for phospholipid class, fatty acid class, and amino acid class (Figure 5c and Figure 6a,b). Differentially expressed genes that played important roles in the biochemical steps of metabolism and synthesis explained some differences in phospholipid metabolites, amino acid metabolites, and fatty acyl metabolites between JXB pigs and DDBJ pigs (Figure 5c and Figure 6a,b). To identify new hub genes most strongly associated with pork quality regulation, differential genes were screened with the criteria foldchange >2 and *p* < 0.05. In total, 444 DEGs were screened (217 upregulated differential genes and 227 downregulated genes; Figure 7a), and to further highlight the possible genes linked to meat quality, we collected data from the PubMed database with the keywords “pig” + “meat quality” + “gene name” (using the selected 444 genes of interest) and identified 56 differential genes. Subsequently, these 56 differential genes were subjected to Spearman correlation analysis with differential metabolites, and the results were visualized as a network plot (Figure 7b). The downregulated genes *CHODL* and *DBX1* and upregulated genes *FLNC* and *MAPK10* were selected according to the absolute value of correlation coefficient |r| > 0.8 and *p* < 0.05 (Figure 7c).

## 4. Discussion

The aim of the present study was to estimate the meat quality of Chinese local pigs and their hybrid pigs. Furthermore, we performed a comprehensive analysis of the LD muscle quality of JXB and DDBJ pigs by combining transcriptome and metabolome analysis, several DEGs and metabolic regulatory pathways related to meat quality were identified, which explained the regulatory mechanism of meat quality differences between the two pig lines. The DEGs regulatory map was drawn according to the omics data, and key functional genes such as *DBX1*, *CHODL*, *FLNC*, and *MAOK10* were mined.

Gene selection has made a significant contribution to the performance improvement of production animals. Notably, genetic improvement may be achieved through intravarietal selection (i.e., pure breeding) and cross breeding (i.e., using native or foreign genetic resources). Hybridization, in contrast with pure breeding, does not lead to genetic advancement (that is, the average value of the parental varieties is equivalent to the additive genetic benefit of hybrid animals) but may provide a variety of benefits, including the use of complementary features and heterosis effects [15]. Many studies have shown that cross-breeding can change pig meat quality traits, carcass traits, nutritional value, energy metabolism, and related gene expression [16]. Although Chinese purebred pigs typically exhibit good meat quality, BQ pigs produced by crossbreeding Bactrian terminal boars with native pigs (Qing Yu) show higher water-holding capacity [17]. In a previous study, pigs with intermediate features in most examined traits such as meat color, intramuscular fat, PH24, and back fat thickness were produced by crossing two local breeds with notable variances in several markers of carcass and meat quality traits [18]. Thus, crossbreeding remains an important option for improving the production of animal traits.

Phospholipids are the only intramuscular lipids that undergo pronounced changes during processing, and when the dry-cured ham is being processed, they serve as the primary substrate for fat decomposition, which may negatively impact the product’s quality [19]. Due to their high polyunsaturated fatty acid content, phospholipids are also vital substrates for lipid oxidation [19]. It is worth noting that to some extent, rancidity is caused by triglycerides and phospholipids [20]. In particular, phospholipids are the first molecules to undergo oxidation, and their contribution to rancidity is by far the most significant. The degree of unsaturation in triglycerides determines the extent to which they contribute to the rancidity of meat. The oxidation of polyunsaturated fatty acids is inextricably linked to rancidity. *DGKA* can phosphorylate diacylglycerol (DAG) to form phosphatic acid, which is the precursor of glycerol phospholipid synthesis [21]. *LIPG* is a cell surface lipoprotein that can help with cell internalization of surface lipoprotein particles (HDL/LDL/VLDL) through endocytosis, leading to their decomposition. In addition, *LIPG* is capable of hydrolyzing high-density lipoprotein that is attached to the cell surface, resulting in the release of lipid precursors like *LPE*, *LPC*, and fatty acids [22]. *LPIN1* and *LPIN3* are involved in the generation of DAG from phosphatic acid [23] *LPCAT4* promotes phosphatidylcholine (PC) to produce lysophosphatidylcholine [24]. Lysophosphatidylglycerol acyltransferase I (*LPGAT1*) is an sn-1 specific acyltransferase, which regulates the stearic acid/palmitic acid ratio of phosphatidylethanolamine (PE) and phosphatidylcholine and prefers stearyl coenzyme A to palmitoyl coenzyme A as the substrate [25]. The lack of *LPGAT1* leads to the substitution of stearate in phospholipids (including PE, dimethylphosphodiethanolamine, and PC) of the PE methylation pathway by palmitate [25]. Two fatty acids are identified in phospholipids and are bound to the glycerol sn-1 and sn-2 carbons [26]. Generally, saturated fatty acids, primarily palmitate (C16:0) or stearate (C18:0), are found at the sn-1 position. Furthermore, sn-2 mostly consists of unsaturated fatty acids, the composition of which is controlled by the process of fatty acid remodeling [27]. 3, Glycerol phosphate acyltransferase (*GPAT*) catalyzed the first step of de novo synthesis of triglycerides, and *GPAT3* was identified as a triacylglycerol biosynthesis enzyme [28]. The different expressions of these up and down differentially expressed genes in DDBJ pigs and JXB pigs revealed the potential gene regulation mechanism of the difference in phospholipid content in their muscles.

Dietary saturated fatty acids may play a role in elevating human blood cholesterol content, which in turn raises the risk of developing cardiovascular disease [5]. When determining the nutritional and health benefits of meat, the ratio of polyunsaturated fatty acids to free fatty acids in the meat is often regarded as the most important factor to consider [29]. Meat that has a sufficient ratio of n-3 to n-6 fatty acids is one way to increase one’s consumption of the healthful n-3 fatty acids via one’s diet, and this may lead to an overall improvement in health. As per previous research findings, n-3 polyunsaturated fatty acids, also known as PUFAs, may increase antioxidant capacity, insulin resistance, and the nutritional value of meat [30]. ALA (C18:3n3) is an essential fatty acid for humans. Other PUFAs can be synthesized through several desaturation extension reactions, such as docosahexaenoic acid (C22:6n3) and EPA (C20:5n3) (M. Xu et al., 2022). C20:5n3 and C20:6n3 can reduce the risk of coronary heart disease. N-6 fatty acids have not been studied as extensively as n-3 fatty acids, and many of the conclusions that have been drawn about them are still up for debate. Fatty acid extentase 5 (*ELOVL5*) is a key enzyme for the endogenous synthesis of long-chain unsaturated fatty acids. It catalyzes the formation of n-3 and n-6 PUFA and is involved in the de novo synthesis of monounsaturated fatty acids [31]. Supplemental PUFA may prevent the fatty tissue deterioration caused by *ELOVL5* ablation (C20:4, n-6; DHA (C22:6, n-3)), as *ELOVL5* is essential for the production of these PUFAs [32,33]. *ELOVL4* (fatty acid elongation enzyme 4) is an endoplasmic reticulum-localized enzyme that facilitates the condensation process during the biosynthesis of C28-C38 ultra long-chain PUFAs (VLC-PUFA) and C28 and C30 saturated fatty acids (VLC-FA) [34,35]. However, *ELOVL5* was upregulated in JXB pigs, and *ELOVL4* was upregulated in DDBJ pigs, which explained why the SFA and PUFA (C < 24) content of JXB pig tissue was higher than that in DDBJ pigs.

*LDLR* is a crucial regulator of cholesterol metabolism that affects the level of circulating low-density lipoprotein cholesterol (LDLc), and it is involved in the clearance of LDLc [36]. *LDLR* is significantly upregulated in JXB pigs, which explains why the cholesterol content in LD muscle tissue of DDBJ pigs was considerably elevated in contrast with that in JXB pigs. *ACAT2* reduces *LDLR* levels and disturbs the cholesterol metabolism homeostasis, thereby inhibiting the differentiation of preadipocytes in pig muscle tissue [37]. *ACAT2* gene expression was significantly upregulated in JXB pigs; thus, we speculated whether inhibition of *LDLR* by *ACAT2* could offset the high expression level of *LDLR* in JXB pigs. Whether *ACAT2* can reduce *LDLR* levels needs further verification. In addition, cholesterol acyltransferase-1 (*ACAT1*) is localized in the mitochondria, which helps form cholesterol lipids from free cholesterol. Cholesterol lipids accumulate in the endoplasmic reticulum [38,39]. Compared with DDBJ pigs, *ACAT1* was also highly expressed in the LD tissue of JXB pigs, which further explains the significant difference in cholesterol content in the LD muscle of the two pig breeds. The downregulation of *CPT2* will cause the β-oxidation process to be inhibited, and the accumulation of acylcarnitine was a surrogate marker for the downregulation of *CPT2* [40]. The *CPT2* was considerably downregulated in the LD of JXB pigs, but more types of acylcarnitine metabolites were downregulated in the LD tissue of JXB pigs, which may require further absolute quantitative tests of acylcarnitine metabolites for confirmation.

Muscle protein quality is determined by the specific amino acid composition of muscle tissue. Generally, three primary processes are involved in the decomposition of proteins: fibrin breakdown from major muscle, polypeptide synthesis as substrates for peptidase, and free amino acid synthesis [41]. Numerous endogenous muscle proteolytic enzymes may be involved in the hydrolysis of meat protein, like calpain, dipeptidyl peptidase, cathepsin, and aminopeptidase [42]. Some free amino acids are considered precursors of flavor substances and can form flavor substances with soluble reducing sugars like fructose and glucose. Apart from alanine, the content of the other 16 amino acids in identified pig LD tissue was remarkably elevated in JXB as opposed to DDBJ pigs. Two distinct subfamilies are discernible within the solute carrier family 7 (*SLC7*): L-type amino acid transporters (*LAT*), comprising *SLC7A15* and *SLC7A5-13*, and cationic amino acid transporters comprising *SLC7A14* and *SLC7A1-4*. We found that the expression of the *SLC7A1* gene was remarkably upregulated in the LD of JXB pigs, and the expression of the *SLC7A2* and *SLC7A4* genes was remarkably upregulated in the LD of DDBJ pigs. *SLC7A1* can perform a major role in the transport of cationic amino acids in pig muscle tissue, and its high expression in JXB pigs may explain why the levels of its cationic amino acids (lysine, arginine, and histidine) were higher than those in DDBJ pigs. Glutamate transporters play an important role in terminating excitatory neurotransmission and providing glutamate for systemic cells for metabolic purposes [43]. The high level of glutamate found in the livers of JXB pigs may be attributed to the high expression level of the *SLC1A1* gene, which encodes the high-affinity glutamate transporter *EAAC1*. The level of *GLUL* gene expression in the LD of JXB pigs was remarkably elevated in comparison to that of DDBJ pigs. The glutamine synthase encoded by the gene *GLUL* converts glutamate and ammonia into glutamine [44]. Therefore, the content of glutamine in the LD of JXB pigs was higher. In addition, we found that some genes involved in the synthesis and metabolism of amino acids, the tricarboxylic acid cycle, and other pathways were significantly upregulated in the LD of JXB pigs, such as *ME2*, *SDS*, and *ALDH18A1*. These differential genes further explained the difference in amino acid content in LD muscle tissue between the two breed lines.

Muscle fiber characteristics and purine content also affect the taste and nutritional value of meat. For example, pig muscle with a higher proportion of large IIB fiber is tougher, lighter, and better marbling score than muscle with a higher proportion of small or normal IIB fiber [45]. Purine metabolism allows for the absorption and use of dietary purines, particularly bases, nucleosides, and nucleotides within the human body. Uricase can catalyze the oxidation of uric acid to allantoin. Humans with attenuated uricase activity have elevated uric acid levels in their blood [46]. Consequently, uric acid is the end product of the purine metabolic process, and once it has been produced, it cannot be decomposed further in the human body. Hyperuricemia and gout are two conditions that may be easily brought on by having a high uric acid concentration in the blood [47]. Therefore, low-purine pig breeds would be less detrimental to consumer health. In addition, we found that eritadenine was downregulated in JXB pigs, although the difference was not significant. Eritadenine had a hypolipidemic effect. Numerous studies examined hypolipidemic activity and the mechanism of eritadenine. However, considering the 160 million hyperlipidemic patients in China, comparatively few studies assessed the application of eritadenine. Therefore, further research and development of eritadenine have a broad market prospect.

## 5. Conclusions

In this study, the abundant metabolic pathways and DEGs were identified based on the multi-omics analysis. These results provide insight into the specific molecular mechanism that regulates meat quality. With further functional verification, these important DEGs may be used to make accurate predictions about the quality of the meat and to guide decision-making in slaughterhouses. Optimizing the composition of fatty acids, phospholipids, amino acids, and other compounds in pork is conducive to improving meat quality. Overall, these findings will give useful information and further groundwork for enhancing the meat quality that may be achieved via hybrid breeding.

## Data Availability

The datasets generated for this study are available on request to the corresponding author.

## Supplementary Materials

The following supporting information can be downloaded at: https://www.mdpi.com/article/10.3390/foods12173237/s1, Figure S1: The carcass traits comparison of DDBJ and JXB; Figure S2: DEGs enrichment classification of KEGG pathway and GO enrichment analysis; Figure S3: Grouping principal component analysis and differential metabolite classification; Figure S4: Differential metabolite enrichment analysis of KEGG, HMDB functional annotation and enrichment analysis; Table S1: Ingredients and nutrients of the basal experiment diets.

## Abbreviations

DLY: Duroc × Landrace × Yorkshire; JXB: Jiaxing black; DDBJ: Duroc × Duroc × Berkshire × JXB; DBJ: Duroc × Berkshire × JXB; BJ: Berkshire × JXB; LD: longissimus dorsi muscle; DEGs: differentially expressed genes; IMP: inosine monophosphate; QTL: quantitative trait loci; UPLC: ultra-performance liquid chromatography; GO: Gene Ontology; EAA: essential amino acids; non-EAA: NEAA; PCA: principal component analysis; LPC: lysophosphatidylcholine; LPE: lysophosphatidylethanolamine; LPA: lysophosphatidic acid; PE: phosphatidylethanolamine; LPGAT1: lysophosphatidylglycerol acyltransferase I; GPAT: glycerol phosphate acyltransferase; PUFAs: polyunsaturated fatty acids; LDLc: low-density lipoprotein cholesterol; LAT: L-type amino acid transporters.
