# Supplementary material for: Multi-Omics Reveals the Effect of Crossbreeding on Some Precursors of Flavor and Nutritional Quality of Pork
Source: Foods. 2023 Aug 28;12(17):3237. doi: 10.3390/foods12173237 (PMC10486348; doi:10.3390/foods12173237)
Supplement: Supplementary file 1 [file foods-12-03237-s001.zip › foods-2566929-supplementary.pdf]

Table S1 Ingredients and nutrients of the basal experiment diets.

Item	20 to 50 kg	50kg to the slaughter
Ingredients(%)		
Puffed corn	30	26
Corn	30	42
Soybean meal	17	10
Puffed soybean	8	18
Whey powder	8	0
fishmeal	3	1
$C_{12}H_{10}Ca_3O_{14}$	1.5	1.2
CaHPO4	1	0.6
salt	0.2	0.2
Lysine	0.1	0.04
Premix	1.2	1
Nutrients		
DE (MJ/kg)	13.70	12.50
CP (%)	18.00	16.00
Ca (%)	0.81	0.65
P (%)	0.68	0.41
Lysine	1.25	0.5

DE = digestible energy; CP = crude protein.

Premix contains the following (unit/kg): 1600 mg of Cu, 10 000 mg of Fe, 3000 mg of Mn, 10 000 mg of Zn, 40 mg of I and 30 mg of Se, 605,000 IU of vitamin A, 155,000 IU of vitamin D3, 1800 IU of vitamin E, 200 mg of vitamin K3, 300 mg of vitamin B1, 400 mg of riboflavin, 200 mg of vitamin B6, 1.5 mg of vitamin B12, 1500 mg of pantothenic acid, 2800 mg of niacin and 12,500 mg of choline.

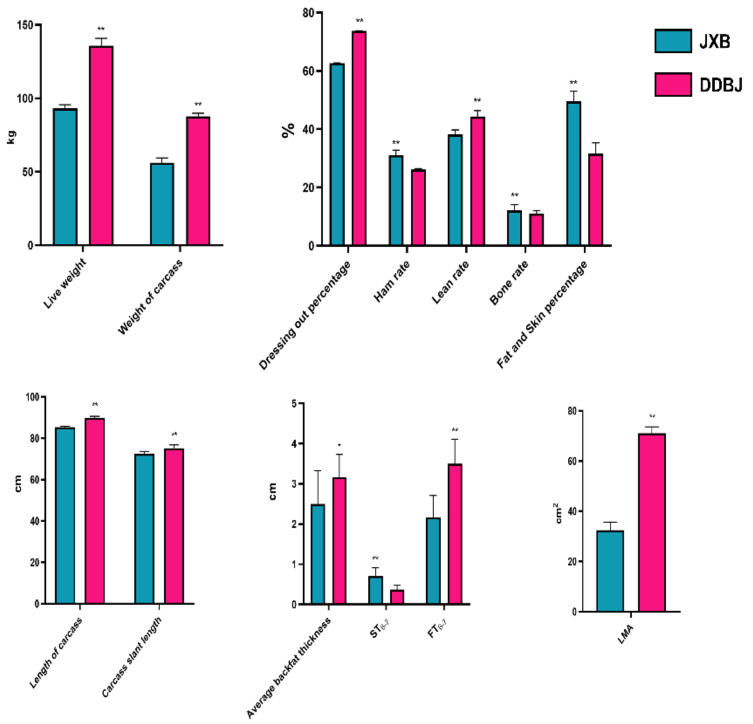

Figure S1. Comparison of carcass traits in DDBJ (n=23) and JXB (n=20) pigs. *indicates a statistically significant difference ($p < 0.05$), ** indicates a highly significant difference ($p < 0.01$); FT6-7 = fat thickness of 6-7th rib of the centerline of the carcass; ST6-7 = Skin thickness of 6-7th rib of the centerline of the carcass.

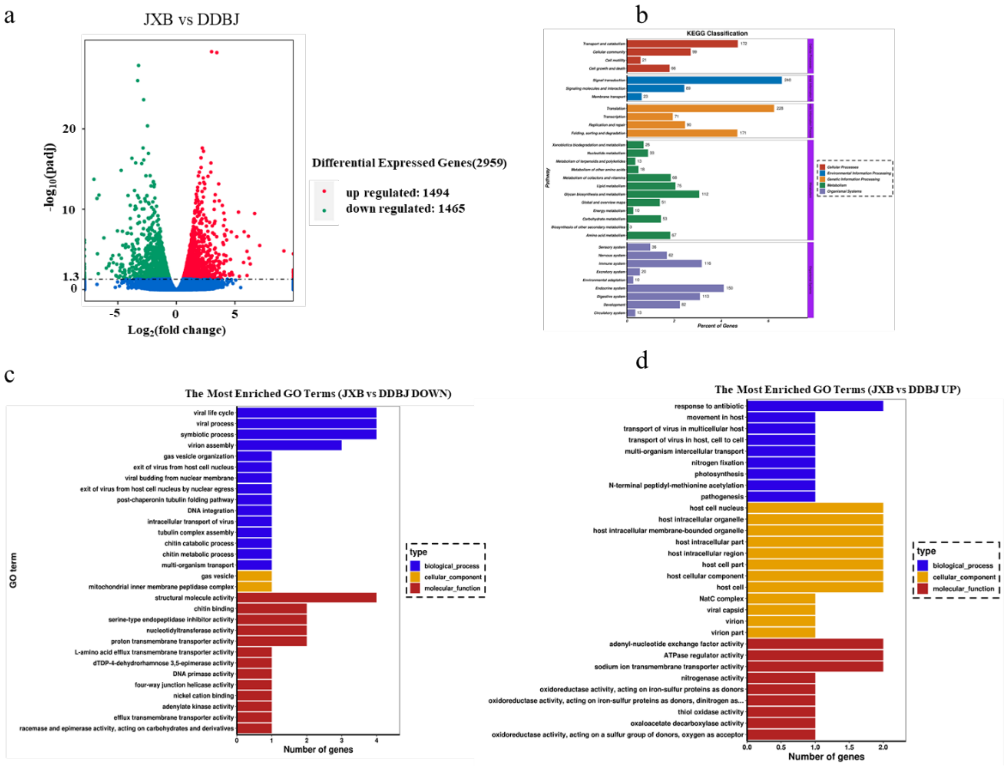

Figure S2. DEGs analysis in LD muscles of DDBJ and JXB pigs. a. Differential expression gene screening volcano map. The screening criteria were: qvalue<0.05. b. Enrichment classification of KEGG pathway. c, d. Up and down-regulate gene GO enrichment analysis histogram.

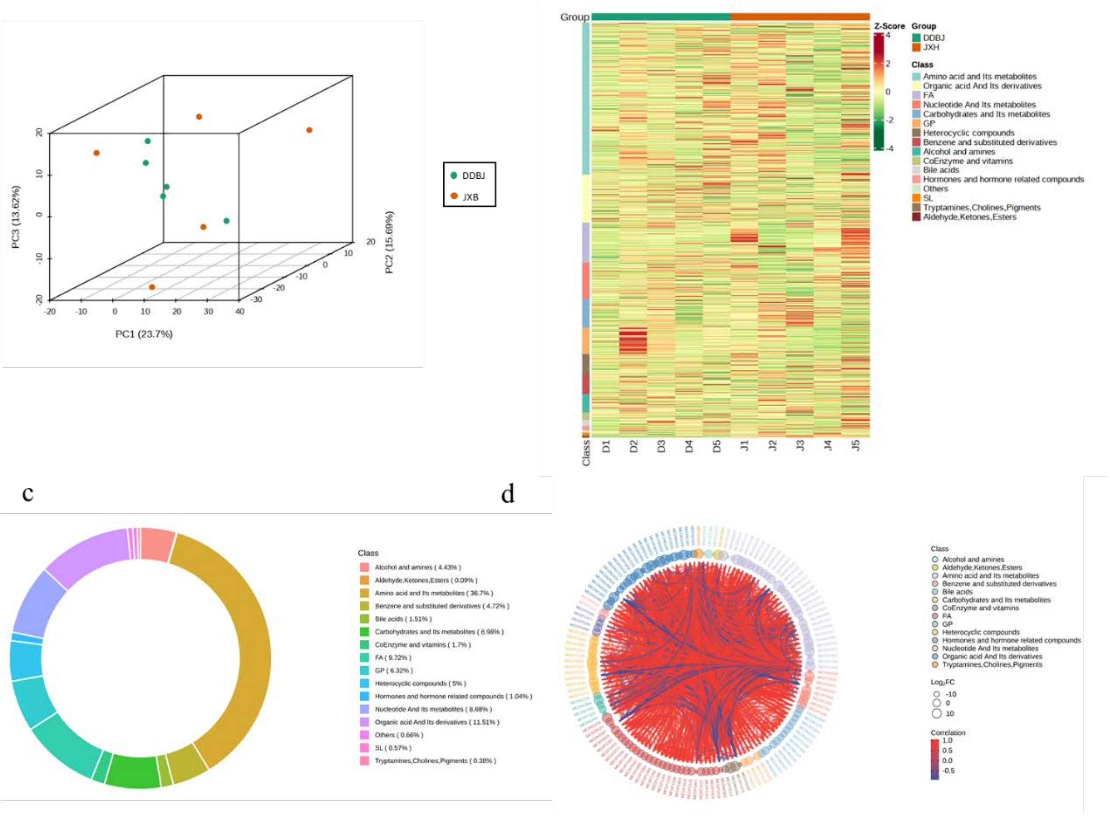

Figure S3. Grouping principal component analysis and differential metabolite classification. a. PCA 3D results. Note: PC1 represents the first principal component, PC2 represents the second principal component, and PC3 represents the third principal component. b. Cluster thermogram of differential metabolites. The horizontal is the sample name, the vertical is the differential metabolite information, the Group is the grouping, and the different colors are the colors filled with different values obtained after the standardization of different relative contents (red represents high content, green represents low content). c. Metabolite categories form a ring chart. Each color represents a metabolite category, and the color block area represents the proportion of this category. d. Differential metabolite and chord diagram. The outermost layer in the diagram is the name of the metabolite, and the size of the point represents the Log2FC value of the corresponding differential metabolite, different colors represent different classifications of corresponding different metabolites, the connecting line represents the Pearson correlation coefficient between corresponding differ ential metabolites, the red line represents positive correlation, and the blue line represents negative correlation. Map the differential metabolite pairs of | r |>0.8 and p<0.05.

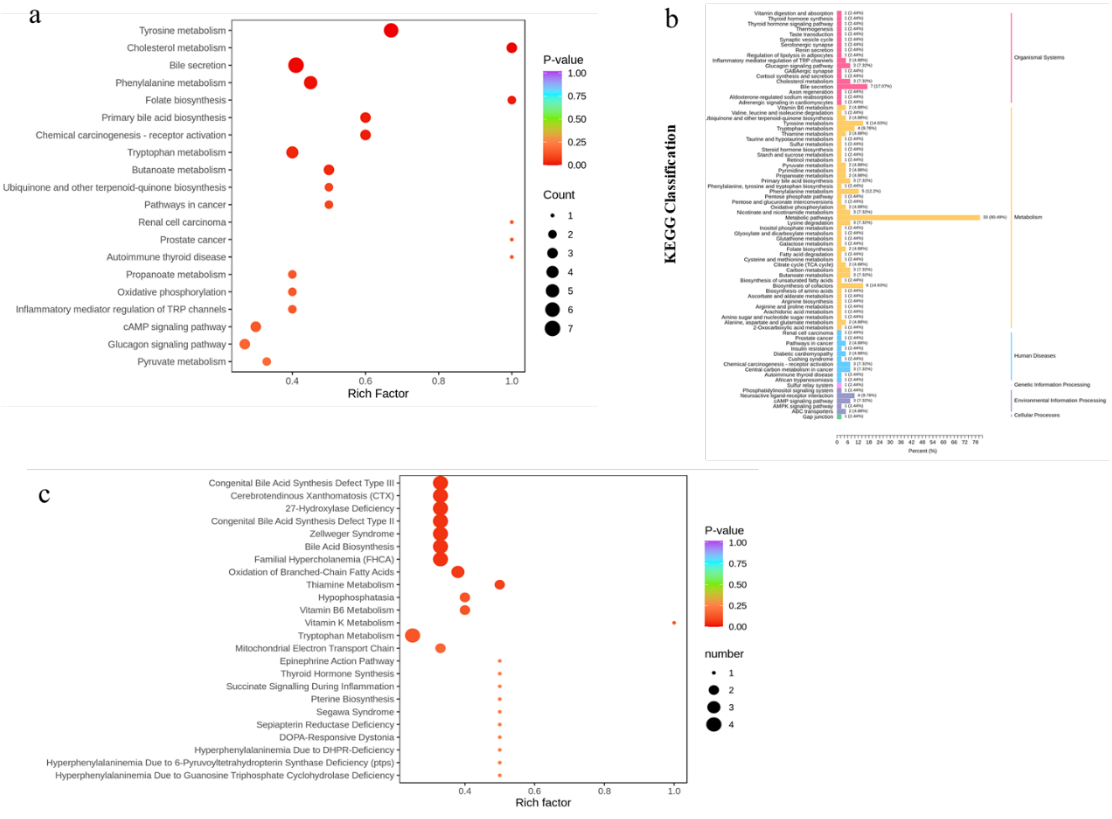

Figure S4. Enrichment analysis of KEGG, HMDB functional annotation and enrichment analysis. a. Enrichment diagram of differential metabolite KEGG. The abscissa represents the Rich Factor corresponding to each pathway, and the ordinate represents the pathway name. The color of the point reflects the P-value size. The redder the point, the more significant the enrichment. The size of the dot represents the number of differential metabolites enriched. b. Classification diagram of KEGG differential metabolites. The ordinate is the name of the KEGG metabolic pathway, and the abscissa is the number of differential metabolites annotated to the pathway and its proportion to the total number of differential metabolites annotated. c. HMDB enrichment map of differential metabolites.
